# Neural Activity Patterns in Response to Interspecific and Intraspecific Variation in Mating Calls in the Túngara Frog

**DOI:** 10.1371/journal.pone.0012898

**Published:** 2010-09-22

**Authors:** Mukta Chakraborty, Lisa A. Mangiamele, Sabrina S. Burmeister

**Affiliations:** 1 Department of Biology, University of North Carolina, Chapel Hill, North Carolina, United States of America; 2 Curriculum in Neurobiology, University of North Carolina, Chapel Hill, North Carolina, United States of America; Rutgers University, United States of America

## Abstract

**Background:**

During mate choice, individuals must classify potential mates according to species identity and relative attractiveness. In many species, females do so by evaluating variation in the signals produced by males. Male túngara frogs (*Physalaemus pustulosus*) can produce single note calls (whines) and multi-note calls (whine-chucks). While the whine alone is sufficient for species recognition, females greatly prefer the whine-chuck when given a choice.

**Methodology/Principal Findings:**

To better understand how the brain responds to variation in male mating signals, we mapped neural activity patterns evoked by interspecific and intraspecific variation in mating calls in túngara frogs by measuring expression of *egr-1*. We predicted that *egr-1* responses to conspecific calls would identify brain regions that are potentially important for species recognition and that at least some of those brain regions would vary in their *egr-1* responses to mating calls that vary in attractiveness. We measured *egr-1* in the auditory brainstem and its forebrain targets and found that conspecific whine-chucks elicited greater *egr-1* expression than heterospecific whines in all but three regions. We found no evidence that preferred whine-chuck calls elicited greater *egr-1* expression than conspecific whines in any of eleven brain regions examined, in contrast to predictions that mating preferences in túngara frogs emerge from greater responses in the auditory system.

**Conclusions:**

Although selectivity for species-specific signals is apparent throughout the túngara frog brain, further studies are necessary to elucidate how neural activity patterns vary with the attractiveness of conspecific mating calls.

## Introduction

Choosing a mate is one of the most important decisions that an animal makes. In many species, females make mate choice decisions based on communication signals produced by males. Males convey information about species identity in their signals and females pay attention to this information in order to avoid heterospecific matings, which often fail to produce viable offspring [1], [2]. Communication signals can also provide information that females use to discriminate among conspecifics, which can lead to variation in male mating success. Thus, the evolution of sender and receiver has been an important topic in speciation and sexual selection [3], [4]. Yet, a complete understanding of how selection acts on behavioral responses to mating signals requires understanding the mechanistic basis of signal processing.

One way to address questions about the mechanisms of sexual communication is to study how the brain responds to variation in male mating signals. In some systems, different signal features differentially predict mate choice in species recognition tasks and intraspecific discrimination, suggesting a hierarchical classification process. For example, in frogs and crickets, fine-scale temporal features (e.g., pulse rate) often influence species recognition whereas gross temporal features (e.g., call duration) often influence intraspecific discrimination [2], [5]. In songbirds, patterns of neural selectivity are consistent with a hierarchical classification process: selectivity to conspecific song is apparent in Field L of the forebrain [6], [7], a primary auditory area, whereas selectivity to preferred song types appears to emerge in secondary auditory forebrain areas such as the caudomedial mesopallium and the caudomedial nidopallium [8]–[11]. However, our understanding of the relationship between the neural mechanisms of species recognition and intraspecific discrimination is incomplete.

Túngara frogs (*Physalaemus pustulosus*) are an excellent model for investigating species recognition and intraspecific discrimination. Male túngara frogs and their close relatives produce advertisement calls, known as whines, that females use to locate and identify potential mates. Male túngara frogs can increase the attractiveness of their whines by adding a second note called a chuck. Although the whine is sufficient for species recognition [12], females prefer whines with chucks compared to whines [13]–[15]; however, the number of chucks does not influence the attractiveness of a male's call in most situations [16]. Based on behavioral choice tests, Wilczynski et al. [17] proposed a model by which the “call analysis system” of the túngara frog encodes acoustic signals. According to the model, species-specific spectral components of the whine trigger species recognition while the presence of chucks increases attractiveness by increasing the acoustic energy in the calls [17]. Thus, we predicted that conspecific calls, but not heterospecific calls, would evoke responses in brain regions involved in species recognition and attractive calls would elicit differential responses in at least some of those same brain regions, thereby serving as a potential mechanism to bias behavioral output towards attractive signals. Previous work in túngara frogs has identified parts of the auditory brainstem as potentially contributing to species recognition [18], [19]. In the present study, we extend those results by examining both the auditory brainstem and its forebrain targets, and by subsequently examining the effects of call attractiveness on those same brain regions. In addition, we focused on females because we were interested in acoustically induced neural activity patterns within the context of mate choice.

We first presented reproductively active female túngara frogs with conspecific whine-chucks, heterospecific whines of a congener, *Physalaemus enesefae*, or no sound and assessed neural responses in the auditory brainstem and its forebrain targets by measuring expression of the immediate early gene *egr-1* (also known as *zif268* and *ZENK*). All but three nuclei known to receive auditory projections demonstrated a greater *egr-1* response to the conspecific calls than the heterospecific calls. We then exposed a second group of female túngara frogs to conspecific whines, conspecific whine-chucks, or no sound. We found that the whine-chucks did not elicit greater expression of *egr-1* than the whines in any of eleven brain regions examined, suggesting that the magnitude of the responses in these brain regions do not explain intraspecific differences in attractiveness. Clearly, further studies that use alternative approaches will be required to identify brain regions that contribute to intraspecific discrimination in the túngara frog.

## Methods

The governments of Costa Rica (INV-ACOSA-008-07; ATM-ACOSA-002-07) and Panama (SEX/A-133-07; SE/A-99-07) permitted tissue collection and export, and the University of North Carolina Institutional Animal Care and Use Committee (08-015) approved our experimental procedures.

### Response patterns evoked by interspecific variation in mating calls

We captured female túngara frogs in a mating clasp with males on the Osa Peninsula, Costa Rica in July 2007 between 20:00 and 24:00 h. We released the males and brought the females to the laboratory at the Osa Biodiversity Center where we placed each in a rectangular mesh cage (18 cm×10 cm) inside one of 8 dark acoustic chambers (91 cm×20 cm×30 cm). Each chamber was equipped with a Tivoli Portable Audio Laboratory speaker (Tivoli Audio, Cambridge, MA) that was connected to an M-Audio Firewire 410 8-channel audio playback unit (M-Audio, Arcadia, CA) and a Macintosh computer. After an 11-hour acclimation period, we presented females with conspecific whine-chucks (n = 11), heterospecific *P. enesefae* whines (n = 11), or no sound (n = 8) for 30 minutes followed by 30 minutes of silence. We dispersed treatments across chambers and days. All females in the study remained gravid during the acclimation period and stimulus presentation. We rapidly decapitated females 1 hour after onset of stimuli, a time that corresponds to peak accumulation of acoustically induced *egr-1* mRNA expression [20] and that occurs before habituation of the *egr-1* response is apparent (R. Glaeser, L.A. Mangiamele, S.S. Burmeister, unpublished observation). After decapitation, we opened the skull in order to fix the brains for 10 min in 4% paraformaldehyde before removing them. We then rinsed the brains in phosphate buffered saline for 10 min before freezing them in liquid nitrogen in 2 ml tubes containing Tissue-Tek OCT embedding medium (Sakura, Finetek, Torrance, CA). We kept the brains on dry ice during transportation to University of North Carolina where we stored them at –80° C until further processing.

**Figure 1:**
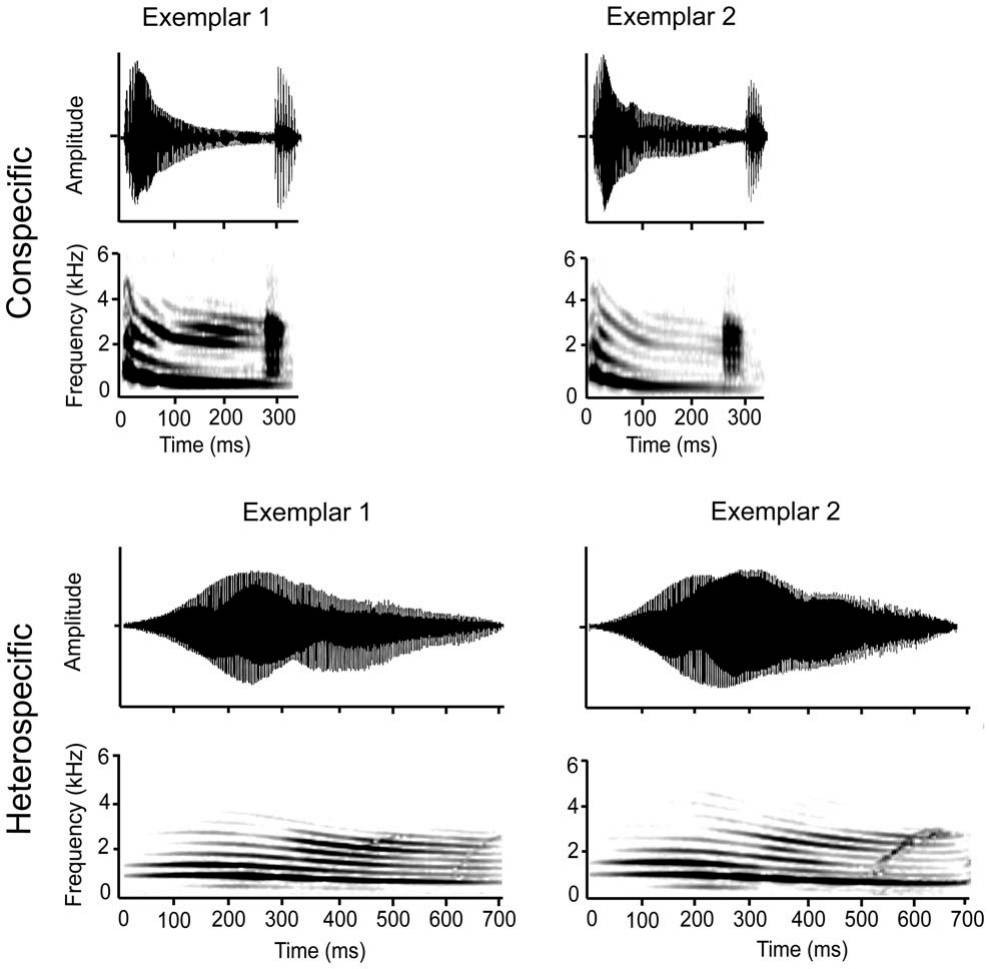
Waveforms and spectrograms of the calls that we used to represent conspecific (*Physalaemus pustulosus*) and heterospecific (*Physalaemus enesefae*) mating calls in order to identify brain regions that contribute to species recognition.

For our initial study on responses to conspecific calls, we chose to use the whine-chuck to represent conspecific calls because it contains both notes in the species's repertoire. We used two túngara frog whine-chucks, each with a single chuck (whine +1 chuck), that we recorded from two free-living males on the Osa Peninsula in 2005 using a digital recorder and a sampling rate of 44.1 kHz (Fig. 1). To represent heterospecific calls, we chose to use the calls of *P. enesefae* because, although they do not occur in Costa Rica, *P. enesefae* is the only congener that is sympatric with the túngara frog and the calls of *P. ensefae* are more similar to those of the túngara frog than more distantly related species that co-occur with the túngara frog in Costa Rica. We used two *P. enesefae* whine exemplars recorded by Dr. Zaida Tárano from two different males in Venezuela (Fig. 1). We presented each female with a single male call that was repeated every 2 seconds in order to reflect the average calling rate of *P. pustulosus* males and to be consistent with previous behavioral [e.g., 16] and *egr-1* studies [e.g., 18]. We set the peak amplitude of the calls at 82 dB SPL (re 20 µPa) at a distance of approximately 5 cm from the speaker with a RadioShack (Fort Worth, Texas) sound pressure level meter.

### Response patterns evoked by intraspecific variation in mating calls

We captured female túngara frogs in a mating clasp near Gamboa, Panama from 29 October to 25 November, 2007 between 20:00 and 22:45 h, transported them to the laboratory of the Smithsonian Tropical Research Institute, and placed each inside a circular mesh arena (8 cm diameter) inside one of 8 dark acoustic chambers (270 cm×190 cm×190 cm). After a 10-h acclimation period, we exposed females to túngara frog whines (n = 9), whine-chucks, each with three chucks (whine +3 chucks; n = 11), or no sound (n = 6) for 30 minutes followed by 30 minutes of silence before sacrifice. We dispersed treatments across chambers and days. We collected the females' brains according to the procedure described above, except that we did not rinse brains in phosphate buffered saline before freezing them.

**Figure 2:**
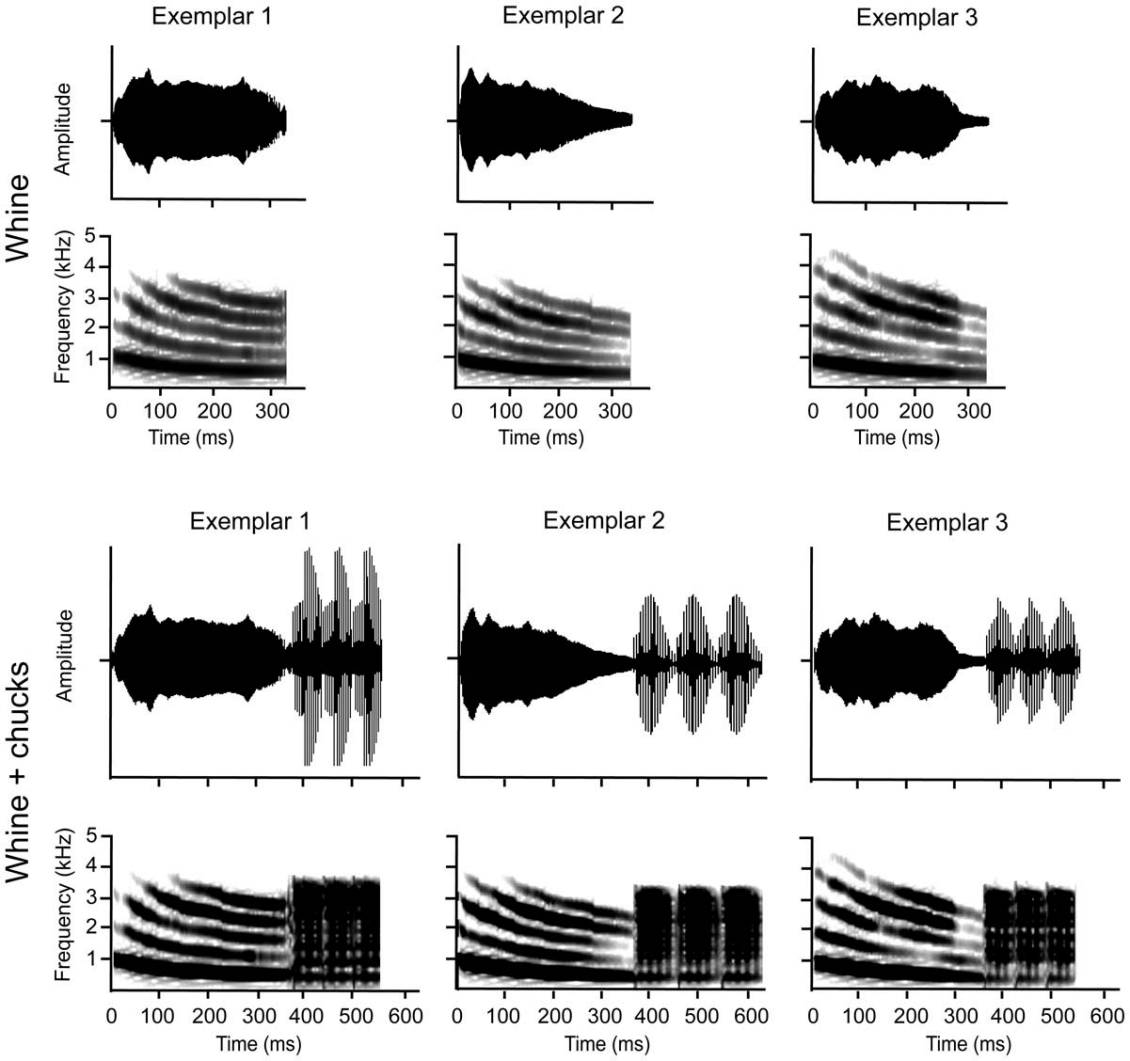
Waveforms and spectrograms of the túngara frog (*Physalaemus pustulosus*) calls that we used to represent the whine and whine-chuck in order to examine responses of brain regions to mating calls that vary in their attractiveness.

To create our stimuli, we started with three whine +1 chuck calls from Ryan and Rand [21], referred to as Oc, M, Sd in the original report, and modified them using Signal sound analysis software (Engineering Design, Berkeley, CA). To create our whine stimuli, we removed the chuck. To create our whine +3 chucks stimuli, we appended the original chuck onto the whine three times. Thus, each experimental group had the same three call exemplars that differed only in the presence of chucks (Fig. 2). Each female heard a single male call that was repeated every 2 seconds to approximate the average calling rate of túngara frog males. In addition, we modified our stimuli to account for the frequency response characteristics of our amplified speaker system by using Vibrotoolbox (Dr. Marcos Gridi-Papp, University of the Pacific) to create a transfer function of our speakers between 100 Hz and 6000 Hz and then filtering each acoustic stimulus by the inverse of this transfer function. We set playback amplitude at 82 dB (re 20 µPa) at approximately 25 cm from the speaker.

### Radioactive *in situ* hybridization

We sectioned brains in the transverse plane at 16 µm in 3 series on a cryostat. To localize *egr-1* mRNA, we used radioactive *in situ* hybridization following the procedure previously described in Burmeister et al. 2008 [20]. Briefly, we generated radioactively labeled (S-35) sense and antisense probes by reverse transcription of a 309-nucleotide subclone of *P. pustulosus egr-1* (GenBank Accession No. AY562993) and hybridized the probes to the brain tissue at 65° C. We performed separate *in situ* hybridizations for brain tissue collected in each experiment. To visualize the bound riboprobe, we dipped slides in emulsion, allowed them to dry, and stored them in lightproof boxes at 4°C for 14 days before development and counterstaining with thionin. To confirm the specificity of our *egr-1* riboprobe, we noted the absence of binding in brain tissue hybridized with sense strand riboprobe under identical hybridization conditions.

### Quantification of *egr-1* expression

**Figure 3:**
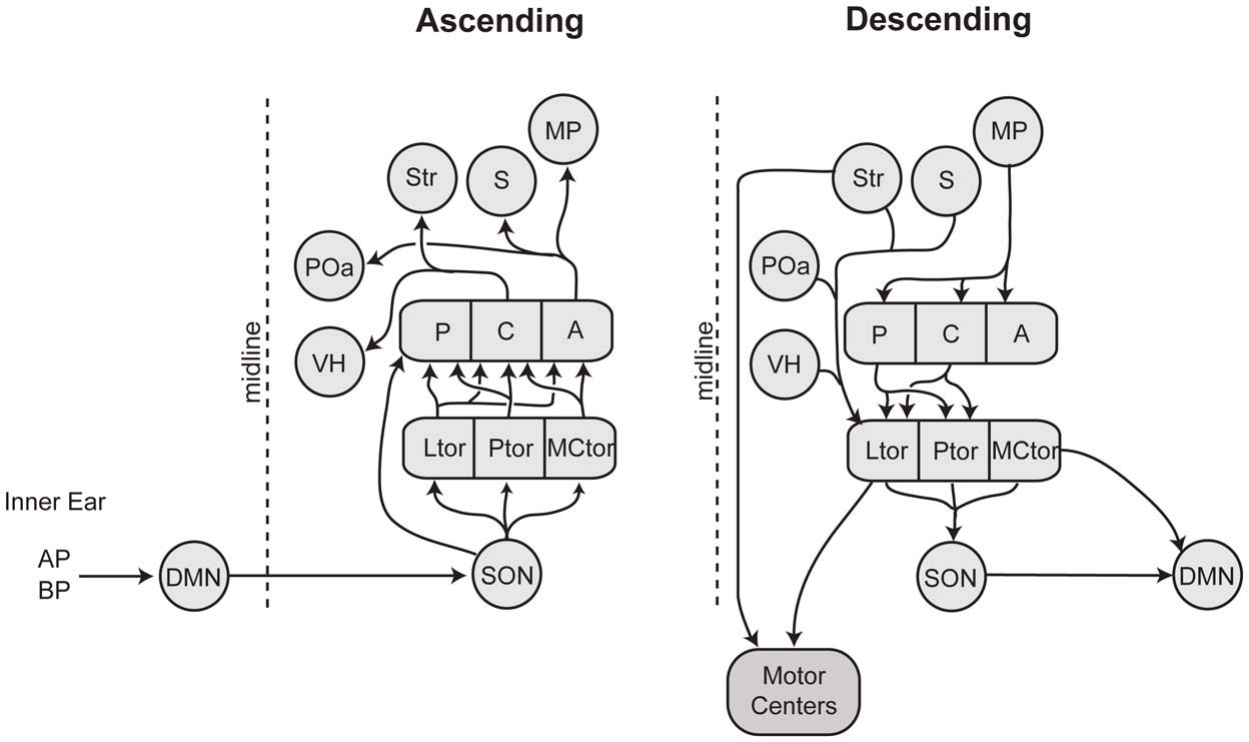
Diagram of the major ascending and descending connections in the frog auditory system. Abbreviations: A, anterior thalamus; AP, amphibian papilla; BP, basilar papilla; C, central thalamus; DMN, dorsal medullary nucleus; Ltor, laminar nucleus of the torus semicircularis; MCtor, magnocellular nucleus of the torus semicircularis; MP, medial pallium; P, posterior thalamus; POa, anterior preoptic nucleus; Ptor, principal nucleus of the torus semicircularis; S, septum; SON, superior olivary nucleus; Str, striatum; VH, ventral hypothalamus.

We measured *egr-1* expression in the auditory brainstem and its forebrain targets (Fig. 3). We examined these regions because of their role in auditory processing [22] or their predicted involvement in female choice behavior in studies of other anurans [23]. The auditory brainstem includes the dorsal medullary nucleus (homolog of the mammalian cochlear nucleus), superior olivary nucleus, and midbrain torus semicircularis (homolog of the mammalian inferior colliculus). Within the torus semicircularis, we sampled from the principal, laminar, and magnocellular nuclei. Forebrain targets of the auditory system include the posterior, central, and anterior thalamic nuclei, the ventral hypothalamus, anterior preoptic nucleus, medial pallium, septum, and striatum. Within the medial pallium, we sampled from the dorsal part. Within the septum, we sampled from the ventrolateral septal nucleus. Within the striatum, we sampled from the ventral portion.

For each brain region, we quantified *egr-1* mRNA expression from digital images taken at a magnification of 630× or 1000× from one hemisphere of the brain chosen at random. Due to variation in tissue quality, we were unable to collect data from all brain regions for every individual; overall, we collected data from an average of 10.6 brain regions per subject resulting in 18–30 subjects per brain region. In addition, the number of sections we sampled from for each brain region varied with the size of the region and quality of the sections, as follows: dorsal medullary nucleus, 2–7; superior olivary nucleus, 2–8; principal nucleus of the torus semicircularis, 3–6; laminar nucleus of the torus semicircularis, 3; magnocellular nucleus of the torus semicircularis, 2–5; posterior thalamus, 3–6; central thalamus, 3–6; anterior thalamus, 2–4; ventral hypothalamus, 3–11; preoptic area, 3–7; ventral striatum, 3–4; ventrolateral septal nucleus, 3–4; dorsal part of the medial pallium, 6. We quantified *egr-1* expression as described in Burmeister et al. 2008 [20]. Briefly, we used ImageJ to count silver grains in the region of interest and in a nearby area of the slide that represented local background silver grain levels. We chose the sampling area for background silver grains by moving the microscope stage until the tissue was no longer visible. This provides an estimate of local background levels that may differ at different positions of the slide due to variation in emulsion thickness. We subtracted the number of background silver grains from the number of silver grains in the region of interest to calculate number of silver grains above background per image. For each image, we manually counted the number of cell bodies in the region of interest from separate photomicrographs. We expressed *egr-1* expression as the number of silver grains above background per cell.

### Statistical Analysis

When examining response patterns evoked by interspecific variation in mating calls, we conducted separate ANOVAs for each brain region in order to test for an effect of stimulus (no sound, heterospecific whine, conspecific whine +1 chuck) on silver grains per cell above background followed by t-tests between pairs of groups. Although brain regions are not independent of one another, we could not account for any covariation among brain regions with a multivariate analysis (e.g., repeated-measures ANOVA) because missing values for individual brain regions would result in the exclusion of most subjects. Because the no sound group does not have exemplars, we could not include exemplar as a factor in our analyses. Therefore, we tested for exemplar effects separately by conducting ANOVAs with stimulus, exemplar, and their interaction as factors, including only the groups receiving calls.

When examining response patterns evoked by intraspecific variation in mating calls, we used ANOVA to test for an effect of stimulus followed by t-tests to compare conspecific calls (whine and whine +3 chucks) to no sound and to compare the preferred, whine +3 chucks to whine for each brain region. In these analyses, we excluded the dorsal medullary nucleus and ventral hypothalamus because *egr-1* expression was not modulated by conspecific calls in these regions in the first experiment (p>0.89 in both cases) or by variation in the attractiveness of the calls in the second experiment (data not shown). Again, we were unable to conduct a multivariate analysis to account for correlated variation across the brain because of missing values for individual brain regions.

## Results

### Response patterns evoked by interspecific variation in mating calls

**Figure 4:**
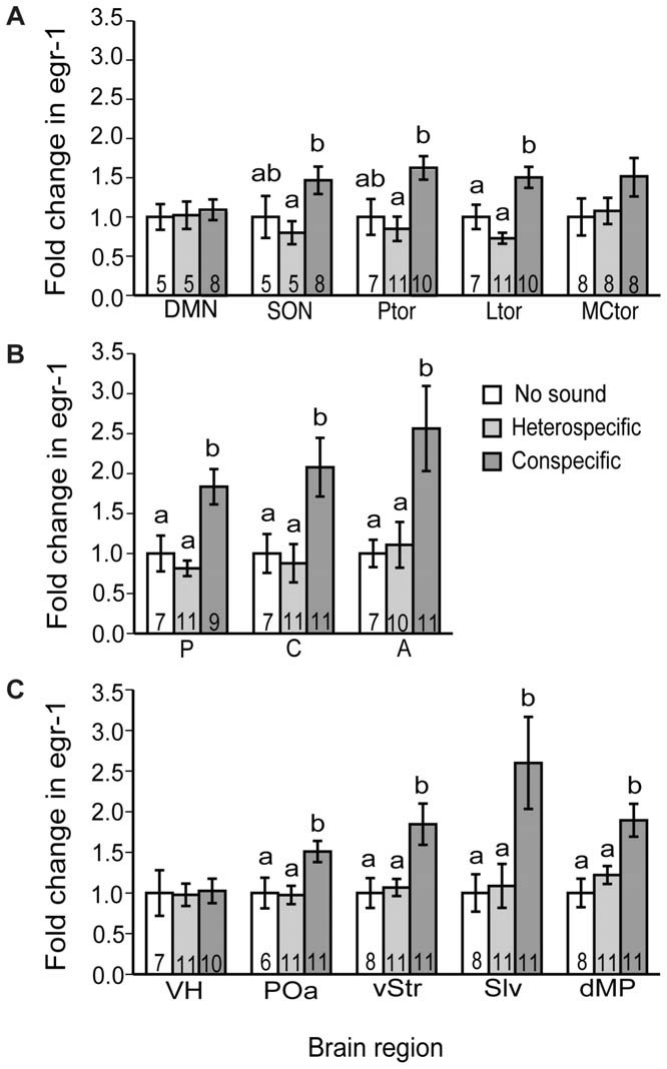
Effects of interspecific variation in mating calls on *egr-1* mRNA expression in the auditory brainstem and its forebrain targets. Data are shown as mean (± SE) fold change in silver grains per cell above background relative to the no sound group. Sample sizes are indicated for each group and letters above bars indicate groups that are statistically different (p<0.05). Abbreviations: A, anterior thalamus; C, central thalamus; DMN, dorsal medullary nucleus; dMP, dosal part of the medial pallium; Ltor, laminar nucleus of the torus semicircularis; MCtor, magnocellular nucleus of the torus semicircularis; P, posterior thalamus; POa, anterior preoptic nucleus; Ptor, principal nucleus of the torus semicircularis; Slv, ventrolateral septal nucleus; SON, superior olivary nucleus; VH, ventral hypothalamus; vStr, ventral striatum.

**Figure 5:**
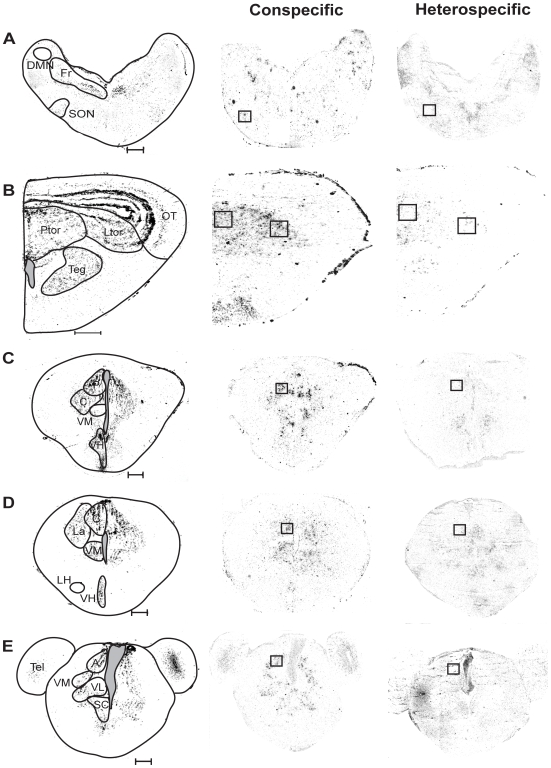
Brightfield images (left column) and inverted darkfield images of transverse sections showing *egr-1* expression within sampling windows (boxes) in response to conspecific whine-chucks (middle column) and heterospecific whines (right column) in the auditory brainstem (A–B) and thalamus (C–E). Scale bars represent 400 µm. Abbreviations: A, anterior thalamus; C, central thalamus; DMN, dorsal medullary nucleus; Fr, reticular formation; La, lateral thalamus; LH, lateral hypothalamus; Ltor, laminar nucleus of the torus semicircularis; OT, optic tectum; P, posterior thalamus; Ptor, principal nucleus of the torus semicircularis; SC, suprachiasmatic nucleus; SON, superior olivary nucleus; Teg, tegmentum; Tel, telencephalon; VH, ventral hypothalamus; VL, ventrolateral thalamus; VM, ventromedial thalamus.

**Figure 6:**
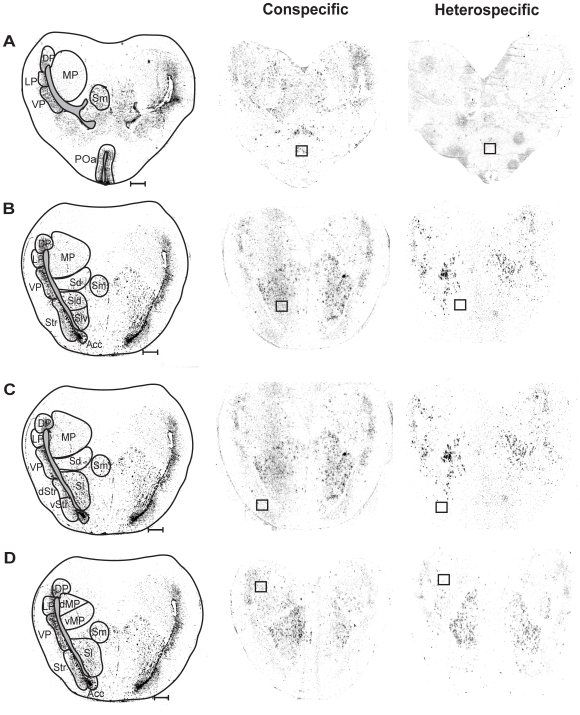
Brightfield images (left column) and inverted darkfield images of transverse sections showing *egr-1* expression within sampling windows (boxes) in response to conspecific whine-chucks (middle column) and heterospecific whines (right column) in the anterior preoptic nucleus (A), septum (B), striatum (C), and medial pallium (D). Scale bars represent 400 µm. Abbreviations: Acc, nucleus accumbens; dMP, dorsal part of the medial pallium; DP, dorsal pallium; dStr, dorsal striatum; LP, lateral pallium; MP, medial pallium; POa, anterior preoptic nucleus; Sd, dorsal septal nucleus; Sl, lateral septal nucleus, Sld, dorsolateral septal nucleus; Slv, ventrolateral septal nucleus; Sm, medial septal nucleus; Str, striatum; vMP, ventral part of the medial pallium; VP, ventral pallium; vStr, ventral striatum.

The conspecific, whine +1 chuck calls elicited robust expression of *egr-1* in almost all nuclei of the auditory brainstem and its forebrain targets while heterospecific whines did not (Table 1; Fig. 4; Fig. 5; Fig. 6). One exception was the dorsal medullary nucleus, where sound had no effect on *egr-1* expression, which was expressed at low but detectable levels. In the superior olivary nucleus, principal nucleus of the torus, and laminar nucleus of the torus, females exposed to conspecific whine-chucks had higher *egr-1* expression than those exposed to heterospecific whines, although they did not always differ from females exposed to no sound, a pattern that reflects a slight decline in *egr-1* expression in females hearing heterospecific whines compared to no sound (Fig. 4). In contrast, conspecific whine-chucks did not have a strong effect on *egr-1* expression in the magnocellular nucleus of the torus. All but one of the auditory forebrain targets we sampled expressed higher levels of *egr-1* expression in response to conspecific whine-chucks compared to heterospecific whines or no sound. In the ventral hypothalamus mating calls had no effect on *egr-1* expression in spite of significant auditory input to this nucleus [24], [25], indicating that sound does not modulate *egr-1* expression there. In no case did exemplar influence the *egr-1* response to calls (exemplar × stimulus, all p>0.13), indicating that the increase in *egr-1* expression in response to the whine-chucks we used may be generalizeable. In summary, conspecific calls elicited significant *egr-1* responses from most parts of the auditory brainstem and its forebrain targets, including limbic and motor regions thought to be important in mate choice.

**Table 1:** Effects of acoustic stimuli on *egr-1* expression when stimuli reflected interspecific (Experiment 1) and intraspecific (Experiment 2) variation in mating calls.

	Experiment 1			Experiment 2		
Region	df	F	p	df	F	p
DMN	2, 15	0.11	0.90	—	—	—
SON	2, 15	3.23	0.07	2,21	4.09	0.03
Ptor	2, 25	6.23	<0.01	2,20	1.46	0.25
Ltor	2, 25	13.34	<0.01	2,22	3.87	0.04
MCtor	2, 23	1.66	0.21	2,19	4.40	0.03
P	2, 24	11.30	<0.01	2,20	1.22	0.32
C	2, 26	6.27	<0.01	2,20	5.82	0.01
A	2, 25	5.06	0.01	2,18	3.89	0.04
VH	2, 25	0.04	0.96	—	—	—
POa	2, 25	5.36	0.01	2,20	3.19	0.06
vStr	2, 27	6.02	<0.01	2,21	1.0	0.38
Slv	2, 27	3.38	0.049	2,22	2.92	0.07
dMP	2, 27	7.71	<0.01	2,22	2.46	0.11

Abbreviations: A, anterior thalamus; C, central thalamus; DMN, dorsal medullary nucleus; dMP, dosal part of the medial pallium; Ltor, laminar nucleus of the torus semicircularis; MCtor, magnocellular nucleus of the torus semicircularis; P, posterior thalamus; POa, anterior preoptic nucleus; Ptor, principal nucleus of the torus semicircularis; Slv, ventral part of the lateral septum; SON, superior olivary nucleus; VH, ventral hypothalamus; vStr, ventral striatum.

### Response patterns evoked by intraspecific variation in mating calls

**Figure 7:**
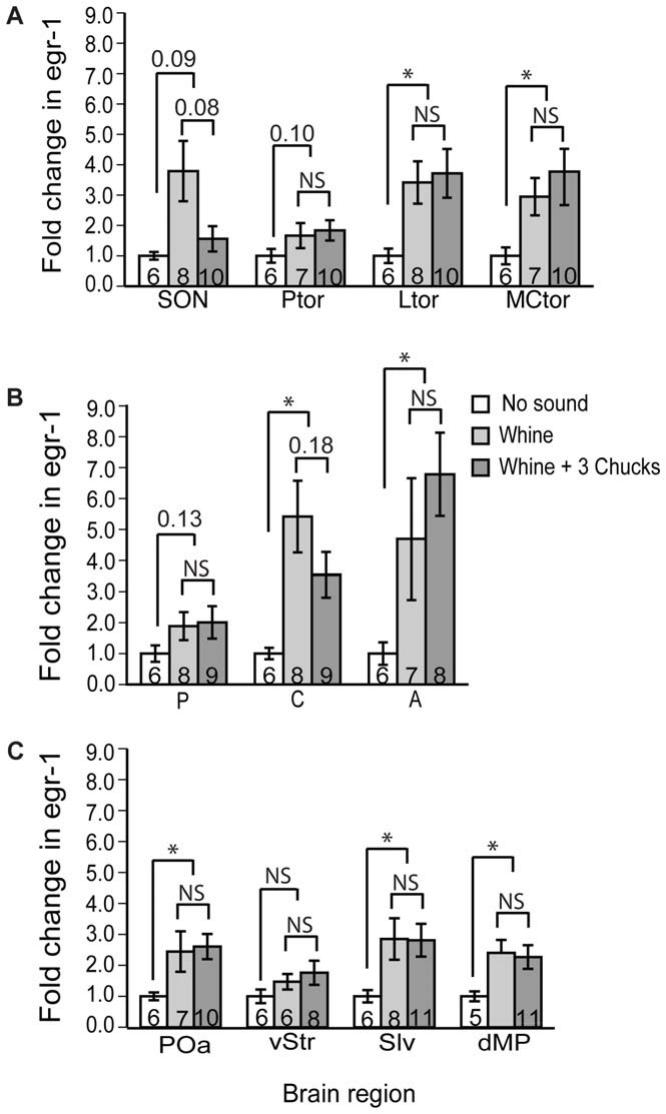
Effects of intraspecific variation in mating calls on *egr-1* mRNA expression in the auditory brainstem and its forebrain targets. Data are shown as mean (± SE) fold change in silver grains per cell above background relative to the no sound group. Sample sizes are indicated for each group. The bars above the columns indicate statistical comparisons between females hearing conspecific calls (whine or whine +3 chucks) to those hearing no sound and between females hearing the preferred, whine +3 chucks to those hearing whines; p values are indicated as follows: asterisks indicate p<0.05, actual p values are given for those tests where 0.05<p<0.2, and NS indicates p>0.2. Abbreviations: A, anterior thalamus; C, central thalamus; dMP, dosal part of the medial pallium; Ltor, laminar nucleus of the torus semicircularis; MCtor, magnocellular nucleus of the torus semicircularis; P, posterior thalamus; POa, anterior preoptic nucleus; Ptor, principal nucleus of the torus semicircularis; Slv, ventrolateral septal nucleus; SON, superior olivary nucleus; vStr, ventral striatum.

Once again, conspecific calls increased *egr-1* expression in most regions of the auditory brainstem and its forebrain targets (Table 1; Fig. 7). However, when we compared conspecific calls (whine and whine +3 chucks) to no sound (Fig. 7), we did not replicate the results from our first experiment (Fig. 4) in three brain regions. For the principal nucleus of the torus, posterior thalamus, and ventral striatum, conspecific calls failed to induce significant expression of *egr-1*, although, because the magnitude of the *egr-1* response in these brain regions was similar in the two experiments, the difference in our results was probably due to differences in the between-subjects variance. In this experiment, however, the *egr-1* response to conspecific calls in the magnocellular nucleus of the torus was more robust than in our first experiment (Fig. 4). We found no evidence for increased *egr-1* responses in females hearing whine +3 chucks compared to those hearing whines in any brain region examined (Table 1; Fig. 7). In all brain regions, levels of *egr-1* expression elicited by whines and whine +3 chucks were similar, with the possible exception of the superior olivary nucleus where the whine appeared to evoke greater *egr-1* expression than the whine +3 chucks. A previous study in males also found no evidence for increased *egr-1* responses to whine-chucks compared to whines in the torus semicirularis [18]. Thus, in spite of robust *egr-1* responses to conspecific calls in most parts of the auditory brainstem and its targets, levels of *egr-1* expression did not vary with the attractiveness of the call.

## Discussion

When evaluating males as potential mates, females must classify them according to species identity and relative attractiveness. To better understand the neural systems underlying these classification tasks, we examined neural activity patterns in response to interspecific and intraspecific variation in mating calls in female túngara frogs by mapping expression of the activity dependent gene *egr-1*. First, we mapped responses to conspecific calls to identify brain regions that contribute to species recognition. Second, we examined responses of these brain regions to mating calls that vary in their attractiveness. We predicted that conspecific calls would differentially stimulate *egr-1* expression in brain regions important for species recognition and that at least some of those brain regions would vary in their *egr-1* responses to mating calls that vary in attractiveness. We found that conspecific whine-chuck calls evoked greater *egr-1* expression than heterospecific whines in auditory, motor, and limbic regions of the brain. However, we found no evidence that preferred whine-chuck calls elicited greater *egr-1* expression than conspecific whines in any brain region examined.

In order to identify neural activity patterns that are characteristic of species recognition, we compared *egr-1* expression in response to conspecific calls to those elicited by the calls of *P. enesefae*, an allopatric congener. Although allopatric species have commonly been used in similar studies [e.g., 19,26,27], there are potential drawbacks. One drawback is that the subjects in all these studies will have had previous experience with the conspecific signals but not the heterospecific ones, resulting in a potential confound between familiarity and species identity (conspecific or heterospecific). Even for species that co-occur, individuals may be more familiar with conspecific than heterospecific signals if they aggregate when signaling, as in the case of many frogs. Nonetheless, at least in the case of túngara frogs, we think that the calls of *P. enesefae* are a useful representative of heterospecific signals because their calls are more similar to those of the túngara frog than other, more distantly related species that co-occur with the túngara frog in Costa Rica. Because phylogenetic relatedness is a good predictor of whether a female will recognize a heterospecific's call as an acceptable sexual signal [28], using a congener to represent heterospecifics is a reasonable approach in this case, even if we cannot rule out the potential contribution of familiarity.

Consistent with previous results [19], we found that differential *egr-1* expression in response to conspecific calls over heterospecific calls emerged as early as the second synapse in the auditory hindbrain, before the emergence of feature detectors in the midbrain and thalamus. Similarly, the auditory midbrain and most of its forebrain targets responded differentially to conspecific mating calls. Surprisingly, the heterospecific *P. enesefae* whine was unable to elicit an *egr-1* response, even though the ears of túngara frogs are sensitive to the spectral content of these calls [29] and behavioral studies confirm that túngara frogs perceive *P. enesefae* calls [30]. Thus, although *P. enesefae* calls are likely to elicit electrical activity in the túngara frog auditory system, they apparently do not activate the second messenger cascades required for induction of *egr-1*, suggesting that *egr-1* responses are more selective than electrical responses in the auditory brainstem. The lack of *egr-1* expression in response to heterospecific calls in túngara frogs is in apparent contrast with zebra finches, where canary song can induce *ZENK* (avian *egr-1*) expression in the auditory forebrain relative to no sound or tones [26].

We found that conspecific calls elicit differential *egr-1* expression throughout the túngara frog brain, including all but three brain regions that receive significant auditory input. In addition to the auditory brainstem, conspecific calls differentially induced *egr-1* in regions of the forebrain that have been implicated in phonotaxis, including the anterior preoptic nucleus, where lesions abolish phonotaxis, the septum, where lesions retard phonotaxis, and the striatum, where lesions abolish locomotion but not orientation [23]. Although the medial pallium, which is homologous to the hippocampus, is acoustically responsive [31], [32], its role in modulating behavior in frogs remains unclear [23]. In spite of the widespread nature of the *egr-1* response, there is reason to think that it is specific to conspecific mating calls. While recordings of a conspecific mating chorus elicit robust responses from the auditory midbrain and some parts of the pallium [20], [31], they do not do so in regions processing other sensory modalities [31], indicating that conspecific mating calls do not elicit *egr-1* responses through some general arousal system. However, we can say very little at this point about the response properties of the different brain regions, as each of them is probably responding to different acoustic traits of the conspecific calls, at least in the auditory brainstem. This point is particularly important in the context of identifying brain regions involved in species recognition since the stimuli we used go beyond those that are sufficient for species recognition in behavioral tests [17]. Whereas we used full-spectrum whine-chuck calls to represent conspecific mating calls, behavioral studies show that the fundamental frequency of the whine is sufficient for species recognition [12] and a sequence of descending tones can mimic the conspecific whine [17]. Future studies that use the minimum required acoustic elements for species recognition would help to elucidate which brain regions contribute to species recognition.

Although the spectral requirements for species recognition by the túngara frog are fairly specific, the requirements for call preferences are highly permissive. In the natural whine-chuck call, the chuck adds acoustic energy in the high frequency range (above 1500 Hz). However, one can emulate the whine-chuck preference with an artificial chuck that contains only the lower frequencies [33]. These types of behavioral studies inspired Wilczynski et al. [17] to propose that mating preferences in the túngara frog result from a simple summation of the acoustic energy in the call. We predicted, therefore, that the whine-chuck call would cause greater *egr-1* expression than the whine in at least some of the brain regions that are responsive to conspecific calls in a manner similar to songbirds where preferred songs elicit greater expression of *ZENK* (avian *egr-1*) in the auditory forebrain [8], [10]. We examined eleven brain regions that were responsive to conspecific calls and none responded to the preferred whine-chuck call with higher expression of *egr-1*. Although not what we expected, our results are consistent with previous studies that also failed to find elevated immediate early gene expression in response to whine-chucks [18], [34]. Interpretation of negative results is always difficult. For example, it is possible that, by presenting stimuli for 30 minutes, we have induced similar levels of *egr-1* expression with both the whines and whine-chucks due to a ceiling effect, rather than a true lack of difference in the calls' abilities to elicit *egr-1* expression. Our interpretation of these results is further limited by the fact that changes in firing rates are not necessarily accompanied by changes in gene expression [35]. Because females show mating preferences for the whine-chuck call, there is no question that the whine-chuck elicits differential responses in the túngara frog brain. We are simply unable to detect these differences using activity dependent gene expression in the manner we have employed to date. At this point, however, we can conclude that our data are not consistent with predictions of the Wilczynski et al. [17] model that posits that mating preferences emerge from greater responses in the auditory system. One limitation of our approach to date is that each female heard only one type of call, whereas mating preferences are a consequence of comparisons among calls. The conspecific whine, by itself, is a very attractive stimulus that is only less attractive in the presence of whine-chucks.

In summary, we mapped neural activity patterns in response to interspecific and intraspecific variation in mating calls in order to better understand the neural mechanisms of mate choice. We found that conspecific calls evoked robust *egr-1* expression in the auditory brainstem and many of its targets, but that preferred, whine-chuck mating calls failed to evoke greater *egr-1* expression compared to whines, in contrast to our predictions based on studies showing that calls of greater acoustic energy evoke behavioral preferences [17]. Clearly we still have much to learn about the neural mechanisms of species recognition and mating preferences in túngara frogs. Because selection for species recognition can influence intraspecific discrimination [36]–[38], determining how the underlying processes are related will enable us to better understand how the evolution of one can influence the evolution of the other.
